# An Easy-to-Handle Route for Bicomponent Porous Tubes Fabrication as Nerve Guide Conduits

**DOI:** 10.3390/polym16202893

**Published:** 2024-10-14

**Authors:** Teresa Russo, Stefania Scialla, Marietta D’Albore, Iriczalli Cruz-Maya, Roberto De Santis, Vincenzo Guarino

**Affiliations:** Institute for Polymers, Composites and Biomaterials (IPCB), National Research Council of Italy (CNR), Mostra d’Oltremare, Pad. 20, V. le J.F. Kennedy 54, 80125 Napoli, Italy; teresa.russo@cnr.it (T.R.); stefania.scialla@cnr.it (S.S.); mariettadalbore@hotmail.it (M.D.); iriczalli.cruzmaya@ipcb.cnr.it (I.C.-M.)

**Keywords:** chitosan, gelatin, porosity, mechanical response, peripheral nerve

## Abstract

Over the past two decades, the development of nerve guide conduits (NGCs) has gained much attention due to the impellent need to find innovative strategies to take care of damaged or degenerated peripheral nerves in clinical surgery. In this view, significant effort has been spent on the development of high-performance NGCs by different materials and manufacturing approaches. Herein, a highly versatile and easy-to-handle route to process 3D porous tubes made of chitosan and gelatin to be used as a nerve guide conduit were investigated. This allowed us to fabricate highly porous substrates with a porosity that ranged from 94.07 ± 1.04% to 97.23 ± 1.15% and average pore sizes—estimated via X-ray computed tomography (XCT) reconstruction and image analysis—of hundreds of microns and an irregular shape with an aspect ratio that ranged from 0.70 ± 0.19 to 0.80 ± 0.15 as a function of the chitosan/gelatin ratio. More interestingly, the addition of gelatin allowed us to modulate the mechanical properties, which gradually reduced the stiffness—max strength from 0.634 ± 0.015 MPa to 0.367 ± 0.021 MPa—and scaffold toughness—from 46.2 kJ/m^3^ to 14.0 kJ/m^3^—as the gelatin content increased. All these data fall into the typical ranges of the morphological and mechanical parameters of currently commercialized NGC products. Preliminary in vitro studies proved the ability of 3D porous tubes to support neuroblastoma cell (SH-SY5Y) adhesion and proliferation. In perspective, the proposed approach could also be easily implemented with the integration of other processing techniques (e.g., electrospinning) for the design of innovative bi-layered systems with an improved cell interface and molecular transport abilities.

## 1. Introduction

The use of porous scaffolds for biomedical applications can be referred back to about three decades ago, and arises from the aim of creating ex novo platforms able to reproduce the microenvironment by mimicking the multiple features of the extracellular matrix (ECM) composing natural tissues [1]. In this view, multiple engineering approaches aimed at designing three-dimensional (3D) systems with high modularity in terms of chemical composition and structural/architectural properties—e.g., porosity, pore size, shape, distribution and orientation, volume pore fraction and interconnectivity—are exploited [2,3,4]. Several works widely debated the pivotal role of the macro-/micro-structure on the ultimate mechanical, mass transfer properties (i.e., nutrient/oxygen diffusion and waste removal) and degradation rate/mechanism (i.e., diffusion and/or erosion) of 3D porous scaffolds that are fabrication process dependent [5,6,7]. In addition, all these features play a relevant role—singularly or synergistically—in influencing cell adhesion and migration, which are essential for triggering tissue growth [8,9]. Hence, it is extremely important to identify biomaterials that combine optimal requirements in terms of biocompatibility/bioactivity and processability to reach the fine control of porous network properties and promote an efficient cell material interaction.

For instance, these engineering aspects can play a key role in the final performance of 3D porous tubes working as nerve guide conduits (NGCs), as an alternative to autologous nerves, to bridge gaps and support regeneration in resected peripheral nerves [10]. NGCs are tubular structures capable of connecting two ends of a damaged nerve—as a result of trauma or a degenerative process—and supporting the oriented growth of axons to recreate the main micro-environmental conditions required for structural and functional nerve regeneration [11]. In this view, an ideal NGC should hold some peculiar requirements in terms of the ability to diffuse nutrients and oxygen to ensure cell activities and metabolism waste before the vascularization, and the biomechanical stability to avoid swelling and mechanical damage to surrounding tissues and axonal regeneration [12]. Moreover, NGCs have to show an appropriate biocompatibility and degradation rate to provide the nerve axons with enough time to grow along the inner surface [13,14]. Several studies exploited multiple manufacturing methods that allow for creating macro-/micro-porosity along the tube wall [4,15]. Chang et al. were among the first to produce porous NGCs by freeze drying a collagen–glycosaminoglycan suspension and exploring the effect of the immersion rate and freezing bath temperature on the resultant pore size (1.5–3.8 mm) and pore orientation [16]. Similarly, Madaghiele et al. implemented a simple technique based on uniaxial freezing and subsequent freeze drying to fabricate collagen-based scaffolds with axially oriented pores intended as NGCs [4]. They found that both the freezing temperature and the collagen concentration significantly affected the mean pore size of the scaffolds. Later, the same group developed a micro-patterned collagen scaffolds by means of a spinning technique that involved thermodynamic and sedimentation phenomena to control the pore size gradient and orientation without the use of any complex mould [17,18]. Tubular conduits with radially oriented pore channels and a radial gradient in the pore sizes were obtained.

According to the previous studies, herein, a simple and versatile approach was explored for the fabrication of NGCs based on chitosan and gelatin, which are widely used to support the interactions with axons, neurons and primary cells [19,20]. Previous studies just verified that chitosan is well-tolerated by the human body, reducing the risk of adverse reactions or inflammation when implanted, and it is also able to gradually degrade over time, avoiding the need for removal through surgery [21]. Moreover, chitosan itself or after chemical modification shows mechanical properties that can adequately support and protect the peripheral nerve during the regeneration process [22]. Accordingly, nerve conduits made of chitosan are rapidly emerging in the market, as confirmed by the recent Food and Drug Administration (FDA) approval for the human use of new products that showed improved performances compared with commercialized collagen devices, which have long been considered as the gold standard for nerve repair [23]. Despite the similarity of several functional outcomes obtained in vivo during the early regeneration period, clinicians still prefer to use autologous grafts to support long-term regeneration for bridging nerve lesions up to 26 mm [24]. Therefore, it becomes of high relevance to further explore new solutions in terms of bioactive materials able to enhance the clinical success of implants, assuring even higher standards of the life quality of patients by the introduction of innovative therapies or the refinement of the more consolidated ones. For this purpose, chitosan was recently hybridized with other naturally derived materials, such as collagen or hyaluronic acid, to validate their use for the treatment of nerve defects [25,26].

In this view, a versatile methodology that is potentially cost-effective compared with alternative technological solutions was proposed to engineer 3D porous chitosan/gelatin tubes with high reproducibility in terms of morphological (e.g., porosity, pore size) and functional (i.e., mechanical) properties. To date, gelatin has been used for the fabrication of nerve conduits, providing adhesive signals able to facilitate axon interactions and limit the formation of thin scar tissue capsules surrounding the conduit in vivo [27], but no works mentioned the opportunity to combine it with chitosan to form 3D porous tubes.

Herein, an accurate optimization of the manufacturing process to fabricate 3D porous tubes with different chitosan/gelatin weight ratios was pursued. The morphological and structural properties and the effects of gelatin addition on the porosity and mechanical features were investigated. A preliminary in vitro study was carried out to confirm the ability of 3D porous tubes to support human neuroblastoma cell (SH-SY5Y) proliferation. Further studies will be undertaken to combine multiple processing techniques toward producing multi-layered systems for nerve guide conduit repair.

## 2. Materials and Methods

### 2.1. Materials

Chitosan (M_w_ 190,000–310,000 Da, deacetylation degree 75–85%, viscosity 200–800 cP for 1% wt in 1% acetic acid at 25 °C—Brookfield); Gelatin Type A (from porcine skin, gel strength 300, Sigma Aldrich, Milan, Italy); and Methyl (1S,2R,6S)-2-hydroxy-9-(hydroxymethyl)-3-oxabicyclo[4.3.0]nona-4,8-diene-5-carboxylate, also named Genipin (GP, ≥98% (HPLC), powder form), were from Merck (Milan, Italy). Glacial acetic acid (ACS reagent, purity ≥ 99.8%), absolute ethanol (EtOH, BioUltra, for molecular biology, ≥99.8%,) and phosphate buffer (PBS) were from Sigma Aldrich, Milan, Italy. Deionized (*di*H_2_O) water (for chromatography LC-MS grade, conductance at 25 °C ≤ 1 μS/cm, Sigma Aldrich, Milan, Italy) was used for the fabrication process and characterization studies.

### 2.2. NGCs Tubular Scaffold Fabrication

**Figure 1:**
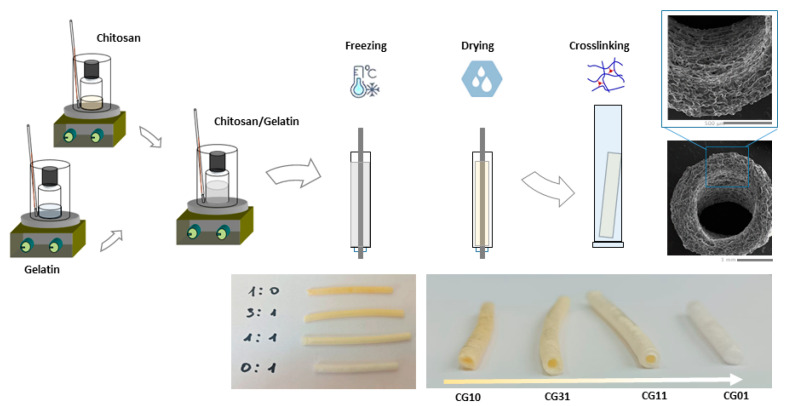
Scheme of NGCs tubular scaffolds fabrication at different chitosan/gelatin ratios by freeze drying.

NGCs tubular scaffolds based on chitosan and gelatin were prepared via the freeze-drying technique, as depicted in Figure 1. Briefly, chitosan (2% *w*/*v*) and gelatin (2% *w*/*v*) were singularly dissolved in acetic acid (2% *v*/*v*) solution via magnetic stirring at room temperature. After 12 h, different volumes of chitosan and gelatin solutions were mixed to reach different chitosan/gelatin (CG) weight ratios—1:0, 1:1, 3:1 and 0:1 *w*/*w* (Table 1). Different CG solutions were vigorously stirred for 1 h until reaching a homogeneous system. Then, CG solutions were one-by-one injected into a tailor-made mold: a 1 mL syringe body assembled with a thin aluminum bar (1 mm in diameter—Figure 1). The system was frozen at −18 °C overnight, and then moved into freeze-dryer equipment (SCANVAC COOLSAFE Labogene Scandivian by Design freeze dryer—Bjarkesvej, Danmark) for three days until obtaining porous structures by means of ice-phase sublimation. Three-dimensional CG porous tubes (Figure 1) were then chemically crosslinked by soaking in 0.4 mM GP aqueous solution for 6 h. The GP solution volume was set to reach 0.5% by weight of GP with respect to the CG weight, according to previous studies [28]. Finally, the samples were stabilized in 10 mM PBS solution for 24 h, washed three times in *di*H_2_O and then gently dried under a hood overnight.

A summary of the process parameters used for the preparation of different 3D CG porous tubes is reported in Table 1.

### 2.3. Morphology

The pore morphology was qualitatively investigated by field-emission scanning electron microscopy (SEM; Quanta FEI 200, Eindhoven, The Netherlands). The NGC tubular scaffolds were cut along the transversal cross-section, coated with an ultrathin layer of Au/Pt by using an ion sputter and analyzed according to previous studies on porous hydrogels [29]. More in detail, 5 mm thick slices were cut with a sharp razor after preliminary soft drying for 3 h under a chemical hood and placed in the observation chamber under low vacuum conditions—0.7 Torr at room temperature.

The 3D structure, as well as the pore architecture, were qualitatively and quantitatively investigated via X-ray computed tomography (XCT) analyses performed via a Nano3DX tomograph (Rigaku Corp., Tokyo, Japan) equipped with a Cr anode that operated at 35 kV, 25 mA. The NGC tubular scaffolds were analyzed by adopting different resolution lens and parameters, as detailed in Table 2.

The volume reconstruction was carried out with the proprietary Nano3DX (CTReconstructionApp, version 2.1.2.0) reconstruction software, as well as adopting ImageJ for image processing of the cross-sections obtained via XCT measurements, according to previous works [30,31,32]. Briefly, the image grayscale thresholds were accurately set to emboss the pore confines, showing the representative space of the pore volume fraction to quantitatively estimate the structural parameters, such as the porosity, pore shape and sizes. The analysis of the aspect ratio, defined as α = a/b, with a and b as the minor and major axes, respectively, was also pursued by assuming that α = 1 (a = b) describes a circular object, whilst α ≈ 0 describes thinner objects. Means and standard deviations were determined from at least five different 3D reconstructions of each scaffold.

The porosity data obtained from XCT elaboration were also compared with those obtained by the gravimetric method, where the porosity (P) was calculated using Equation (1):

(1) $$P=1-\frac{\rho_{a}}{\rho_{m}}$$

where ρ_a_ is the density of the scaffold—calculated as the scaffold weight to the volume—and ρ_m_ is the density of the raw materials—as a function of their relative weight ratio [6].

### 2.4. Physico-Chemical Analysis

Fourier transform infrared spectroscopy coupled with the attenuated total reflectance technique (FTIR-ATR—Perkin Elmer Spectrum 100 FTIR spectrophotometer, Milan, Italy) was used to evaluate the chemical compositions of the porous tubes. Spectra were acquired in the spectral region between 500 and 4000 cm^−1^. The analysis was performed using Origin software (OriginPro 8 SR0; OriginLab Corporation, Northampton, MA, USA).

The ninhydrin assay was adopted to assess the amount of gelatin released by chitosan/gelatin scaffolds. Gelatin (10 mg/mL) was dissolved in PBS (10 mM, pH 7.4) and serial dilutions from 10 to 0 mg/mL were prepared for the standard curve (y = 0.0162x + 0.035, R^2^ = 0.9923). A ninhydrin solution (0.1 g in 100 mL of 70% (*w*/*v*) EtOH) was added to about 3 mg of chitosan/gelatin scaffolds (1 mL/scaffold) in a tube and heated at 80 °C for 20 min. The reaction of ninhydrin with amino groups induced a staining change from white to a blue color in the scaffolds and in the solution as a function of the amount of amino groups. The absorbance of the supernatant was recorded at 570 nm using a UV–Vis spectrophotometer (Victor X3 Multilabel Plate Reader, Perkin Elmer, Italy). The percentage of released gelatin was estimated by removing the contribution of chitosan (CG10) and normalizing with respect to the pure gelatin scaffold (CG01) according to Equation (2):

(2) $$Gelatine\;released\left(\%\right)=\frac{\left({Abs}_{sample}-{Abs}_{CG10}\right)}{{Abs}_{CG01}}\;\cdot 100$$

### 2.5. Water Uptake

NGCs tubular scaffolds at different CG ratios were prepared and the initial dried mass (w_0_) was recorded. Then, the scaffolds were incubated and left to swell in 10 mM PBS solution at pH 7.4 and room temperature until reaching an equilibrium state. At different time points (15, 30, 60 and 120 min), the swollen scaffolds were taken out from PBS and the excess PBS solution was removed with a filter paper. For each scaffold, the weight was recorded (w_s_), and then the scaffold was placed back in the PBS. The water uptake percentage was calculated according to Equation (3):

(3) $$Water\;uptake=\frac{w_{s}-w_{0}}{w_{0}}\cdot 100$$

### 2.6. Mechanical Properties

**Figure 2:**
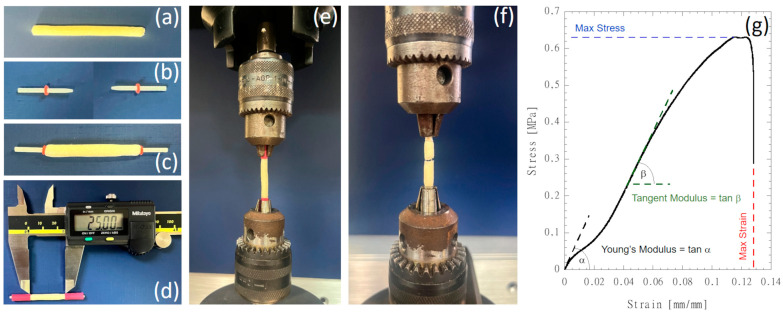
NGC tubular scaffold preparation and mechanical testing: (**a**) tubular scaffolds cut to a length of 45 mm; (**b**) cylindrical glass-fiber-reinforced posts with a length of 12 mm equipped with O-rings positioned at a distance of 5 mm from the tapered end; (**c**) composite posts inserted in the hollow cavity of each scaffold; (**d**) insertion of thermal shrinking poly(ethylene) tubes to fix the posts at both ends of each scaffold and to define the gauge length of 25 mm; (**e**) scaffolds were clamped on the dynamometric equipment through chucks and tensile stretched up to rupture (**f**); (**g**) typical stress vs. strain curve showing the mechanical properties that were investigated.

Mechanical tensile tests were carried out using the Instron dynamometric machine 5566 (Instron, Bucs, UK) equipped with a 10 N loading cell. The NGC tubular scaffolds (external and internal diameters of 4 mm and 1.5 mm, respectively) were cut to a length of 45 mm (Figure 2a). Cylindrical glass-fiber-reinforced posts with a length of 12 mm were equipped with O-rings positioned at a distance of 5 mm from the tapered end (Figure 2b). Composite posts were inserted in the hollow cavity of each scaffold (Figure 2c) to preserve the tubular structure before clamping, and thermal shrinking poly(ethylene) tubes were used to fix the posts at both ends of each scaffold and to define the gauge length of 25 mm (Figure 2d). Each scaffold was clamped on the dynamometric equipment through chucks (Figure 2e), and a preload of 0.05 N was applied before running the tensile test at a speed of 1.0 mm/min up to rupture (Figure 2f).

Force (F) and elongation (DL) data were acquired at a speed of 10 pts/s. The stress was computed by considering the ratio F/A, where A is the specimen’s cross-section area, while the strain was computed through the ratio DL/L_0_, where L_0_ is the initial scaffold length (i.e., 25 mm). Mechanical properties (i.e., Young’s modulus, tangent modulus in the steeper linear region, maximum stress and maximum strain) were detected on the stress vs. strain diagrams, as depicted in Figure 2g.

Analysis of variance followed by Tukey’s post hoc test were performed at a probability level *p* = 0.05 using OriginPro 2018 software (OriginLab Corporation, Northampton, MA, USA).

### 2.7. In Vitro Studies

#### 2.7.1. Cell Line and Chemicals

A human SH-SY5Y neuroblastoma cell line was from ATCC^®^ (HTB-11, cat. no. CRL-2266™). Dulbecco’s Modified Eagle Medium F12 (DMEM/F12 1:1—Gibco™) was from Thermo Fisher Scientific (Monza, Italy). Foetal bovine serum (FBS), L-glutamine (L-Glu, 200 mM), penicillin–streptomycin solution (Pen/Strep, 100×), non-essential amino acids solution (NEAAs, 100×) and trypsin/EDTA solution (1×) were from Sigma (Milan, Italy). Dimethyl sulfoxide (DMSO, ≥99.5%) was from VWR (Milan, Italy). Alamar Blue^®^ was from Bio-Rad Laboratories S.r.l. (Milan, Italy).

#### 2.7.2. Cell Culture Conditions and NGC Tubular Scaffolds Preparation

Immortalized SH-SY5Y cells were adopted as a suitable in vitro cell model for assessing the cytocompatibility of NGC tubular scaffolds. SH-SY5Y cells were grown in DMEM/F12 1:1 supplemented with 10% (*v*/*v*) FBS, 2 mM L-Glu, 1% (*v*/*v*) Pen/Strep solution and 1% (*v*/*v*) NEAAs at 37 °C in a water-saturated atmosphere of 5% CO_2_ and 95% air. The culture media was replaced every two to three days. For propagation, after removing the medium, the cells were washed with 10 mM PBS and detached with a 0.3% (*w*/*v*) trypsin solution. Cell suspension was harvested by centrifugation (1200 rpm, 5 min), and the cell pellet was re-suspended and transferred to new flasks once reaching 70–90% confluence (every 2–3 days).

Before the cell seeding, the NGC tubular scaffolds were sterilized by incubation in 70% (*v*/*v*) EtOH supplemented with 10% (*v*/*v*) Pen/Strep solution for 2 h under ultraviolet (UV) irradiation. Several washes with 10 mM PBS were performed in order to remove the EtOH in excess. Then, the NGC tubular scaffolds were equilibrated in serum-free and serum-supplemented cell culture media for the indirect and direct cytotoxicity assays, respectively, according to ISO 10993-12:2021 [33] for 72 h at 37 °C and 5% CO_2_ under sterile conditions.

#### 2.7.3. Cell Viability

The capability of NGC tubular scaffolds to support SH-SY5Y proliferation was assessed by direct assays. After the sterilization, the NGC tubular scaffolds were equilibrated in 1 mL of cell culture medium prepared as reported in Section 2.7.2 for 30 min at 37 °C and 5% CO_2_ under sterile conditions. Then, the SH-SY5Y cells (1 × 10^4^ cells/scaffold) were directly seeded on the NGC tubular scaffold and left to adhere for 4 h. After 4 h, fresh medium was drop-wise added to each cell-seeded scaffold (200 μL/well). The adhesion capability of the SH-SY5Y cells was quantitatively assessed after 24 h by an Alamar Blue^®^ assay, after moving them to a clean well plate, in order to measure the metabolic activity of the only adhered cells. Meanwhile, the SH-SY5Y cell proliferation was recorded for up to 14 days, where the same method was adopted. Alamar Blue^®^ solution (10% (*v*/*v*)) was prepared in phenol red-free DMEM and it was added to each cell-seeded scaffold and incubated for 4 h at 37 °C and 5% CO_2_. Then, 100 μL of supernatant was transferred to a 96-well plate and the absorbance was measured using a UV–Vis spectrophotometer (VICTOR X3, 156 PerkinElmer, Milan, Italy) at wavelengths of 570 and 600 nm. The cell viability was expressed as a percentage of the Alamar Blue^®^ reduction according to Equation (4):

(4) $$Alamar\;Blue\;reduction\;\left(\%\right)=\frac{\left(O_{600nm}\cdot {Abs}_{570nm}\right)-\left(O_{570nm}\cdot {Abs}_{600nm}\right)}{\left(R_{570nm}\cdot {BLk}_{570nm}\right)-\left(O_{600nm}\cdot {Blk}_{600nm}\right)}\cdot 100$$

where O_570nm_, O_600nm_, R_570nm_ and R_600nm_ are the molar extinction coefficients of oxidized (O) and reduced (R) Alamar Blue^®^ reagent at 570 nm and 600 nm, respectively. Abs_570nm_ and Abs_600nm_ refer to the absorbances of the samples at 570 nm and 600 nm, respectively. Blk_570nm_ and Blk_600nm_ are the absorbances of the negative control well (red-free DMEM medium without cells plus Alamar Blue^®^ reagent) at 570 nm and 600 nm, respectively.

## 3. Results and Discussion

To date, different approaches have been investigated to fabricate 3D porous conduits intended for peripheral nerve regeneration. Most of them have involved the use of collagen for the fabrication of interface materials able to create a suitable microenvironment for nerve regeneration and mimicking the fibrous structure of the ECM of the peripheral nerve [34]. Several studies demonstrated the ability of collagen conduits to guide the Schwann cells’ response and maintain the physiological function of peripheral nerves [35], thus enabling it to obtain FDA approval for clinical trials [36]. Accordingly, all the commercially available nerve guides are currently made of collagen. However, several limitations still concern their therapeutic effects, especially for bridging larger nerve gaps, mainly due to a lack of mechanical properties [37]. Only recently, there has emerged a growing interest for chitosan as an alternative therapeutic material that is highly suitable for repairing nerve defects and promoting nerve regeneration and functional recovery [23]. Indeed, chitosan is a polyelectrolyte with different charge densities as a function of selected features—e.g., acetylation degree, environmental pH—which dramatically influence the chemical stability and degradation properties [38]. Its peculiar ability to work as polycations allows for it to easily interact with natural proteins largely present in the ECM. This offers the unique opportunity to blend it with natural proteins, such as gelatin, and promote the formation of polycation/polyanion complexes by electrostatic interactions between oppositely charged groups along their macromolecules, ensuring improved stability in biological fluids [39].

Herein, we propose the fabrication of bicomponent porous tubes by a versatile approach based on the freeze-drying process of different chitosan/gelatin solutions by using tailorable molds for the fabrication of 3D tubes with a high porosity and controlled geometry. Previous studies that focused on the use of bioresorbable nerve conduits remarked several benefits of chitosan due to its in vitro interactions with growth factors and cytokines, which are able to enhance the proliferation and differentiation of nerve cells and to regulate the neural regeneration process [40]. Other relevant advantages are also related to the in vivo degradation products of chitosan, with the ability to promote axon regeneration—e.g., stimulating Schwann cell division [41], inhibit apoptosis [42] and prevent oxidative stresses [43], and thus, reduce the formation of post-traumatic neuroma and related neuropathic pain [44].

Meanwhile, the addition of natural proteins, like collagen or gelatin, is positively valuable, being able to favor the graft bio-recognition and cell adhesion in the tubular structure by supporting the cell material interactions at the interface with the lumen surface [45].

In this work, chitosan with an average M_w_ and high deacetylation degree (ca. 85%) was chosen to reach a good compromise in terms of the polymer crystallinity and chemical stability [46]. During the preparation of 3D tubes, the amino groups on the chitosan chains dispersed in acidic aqueous solution (pH < 6) tend to be easily protonated, thus promoting their interaction with negatively charged groups of gelatin, as reported in previous studies on similar systems [47]. These peculiar interactions enable the formation of weak bonds among oppositely charged groups of the two different polymers, which promotes a certain stability of the porous structure after freeze drying that is sufficient to prevent any significant alteration of pore features during the crosslinking treatment via genipin [48]. Genipin—a natural water-soluble crosslinking agent isolated from plants that is about 10^4^ times less toxic than glutaraldehyde—was selected due to the ability to interact with both polymers by reacting with the -NH_2_ groups [49].

In this view, the ninhydrin assay was performed to detect the amount of gelatin released by the 3D scaffolds conditioned in the aqueous solution. Figure S1 shows that the chitosan samples—rich in amino groups—tended to be completely stained, while a slight staining of the medium was also recognized. In the case of the gelatin samples, the medium appeared strongly stained due to the interaction of ninhydrin with amine groups in the solution. In the other cases—i.e., chitosan/gelatin scaffolds—the samples were still completely stained and the medium colour was comparable with those of CG10, qualitatively confirming a limited tendency of gelatin to be released. This was corroborated by the absorbance measurements in the medium, with reported released gelatin percentages equal to 12 ± 3% and 19 ± 3% after 20 min for 3:1 and 1:1 chitosan/gelatin, respectively.

**Figure 3:**
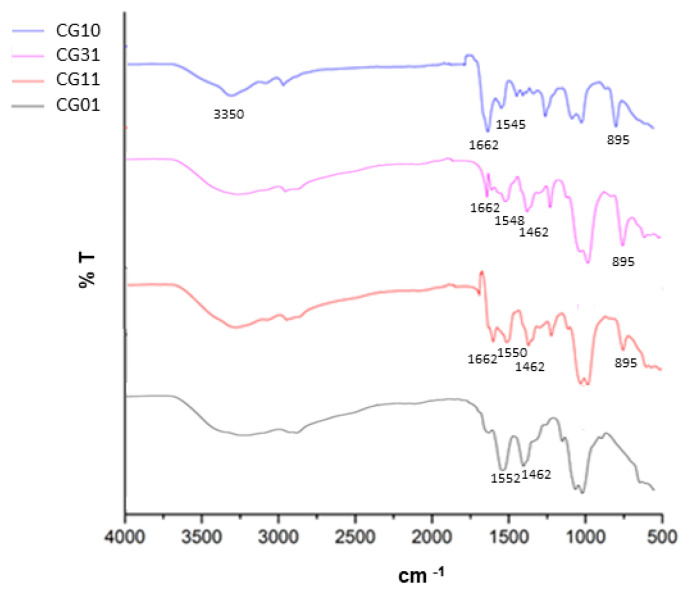
FTIR analysis of tubular scaffolds after the crosslinking treatment as a function of the chitosan/gelatin weight ratio.

The chitosan/gelatin scaffold composition was further investigated after the crosslinking treatment via FTIR. In Figure 3, the FT-IR spectra of bicomponent tubes with different chitosan/gelatin ratios are reported. All the spectra were collected after the crosslinking treatment by genipin.

In the case of CG10, a broad band around 3350 cm^−1^ corresponding to the overlap of N–H and O–H stretching was highlighted [50]. Other characteristic peaks at 1662 cm^−1^ and 1545 cm^−1^ were related to primary and secondary amine groups [51]. Lastly, the peak at 895 cm^−1^ corresponded to the CH bending out of the plane of the ring of monosaccharides. In the case of CG01, the main representative peaks were recognized at 3433 cm^−1^, which was attributed to the presence of hydrogen bond water and amide-A; at 1552 cm^−1^ for amide II (N–H bending vibration); and from 1460 cm^−1^ to 1380 cm^−1^, which was related to the symmetric and asymmetric bending vibrations of the methyl group [52]. For both systems, it was also possible to recognize a relevant peak at 1082 cm^−1^, which is characteristic of the C–N stretching vibration. In the case of bicomponent tubes, the CG31 and CG11 chitosan/gelatin interactions contributed to slightly shifting the characteristic peaks in the range from 1452 cm^−1^ to 1662 cm^−1^—related to C-O and C-N stretching vibration—with structural changes through the Schiff base reaction [51].

**Figure 4:**
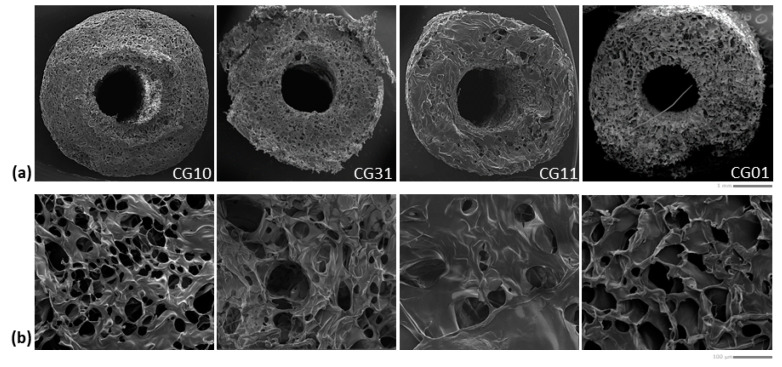
Morphological investigation of the tubular scaffolds with different chitosan/gelatin ratios (1:0, 3:1, 1:1, 0:1): qualitative evaluation of pores along the cross-section (scale bar: 1 mm) (**a**) and surface (scale bar: 100 μm) (**b**).

In Figure 4, the morphological investigation of crosslinked tubes with different chitosan/gelatin ratios is reported.

The SEM images of the sample cross-section (Figure 4a) showed a well-defined tubular geometry of the scaffold with a porous network architecture characterized by pores homogeneously dispersed into the polymer matrix. More remarkable interconnections of pores could be qualitatively recognized in the case of mono-component systems—e.g., CG10 and CG01—as confirmed by the image of the scaffold portions (Figure 4b).

**Figure 5:**
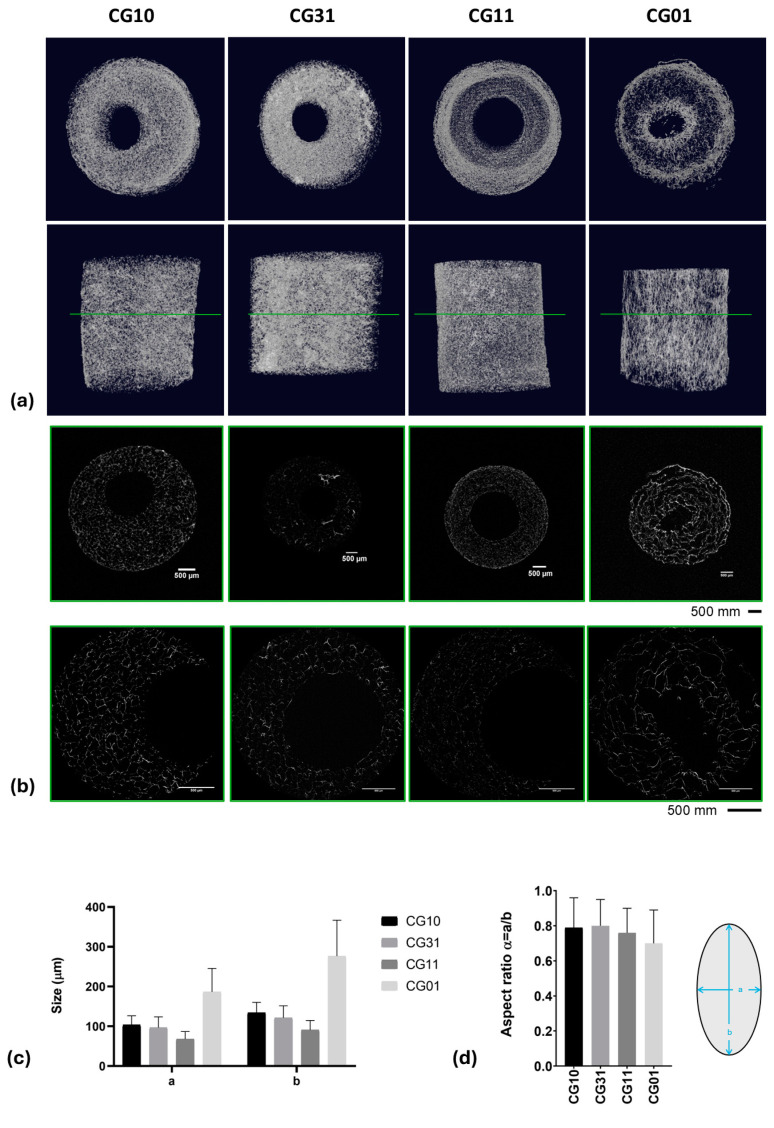
XCT analyses: 3D rendering (**a**) and 2D slicing (**b**). Data obtained from XCT data processing: a and b pore sizes (**c**) and aspect ratios (**d**) reported as the mean value ± standard deviation for the different analyzed 3D scaffolds.

Quantitative information about the 3D scaffold porosity was collected by gravimetric measurements (Table 3) showing a percentage (v) of pores that ranged from 94% to 97% in the cases of chitosan and gelatin scaffolds, respectively. Intermediate values of porosity were also detected for blended scaffolds as a function of the increased gelatin content. Similar trends of porosity were collected on 3D scaffold volumes rendered via XCT analyses (Figure 5a and Table 3).

A highly porous structure with thin walls was observed, consistent with data reported by Grenier et al. on freeze-dried gels [53]. The pore shape analysis performed on thin cross-sections obtained via XCT analyses (Figure 5b) highlighted a spheroidal shape, with the a and b minor and major axes, respectively, reported in Table 3 and Figure 5c. The aspect ratio, defined as α = a/b, highlighted values that ranged from 0.70 ± 0.19 for samples named CG01 to 0.80 ± 0.15 for samples named CG31 (Table 3 and Figure 5d).

**Figure 6:**
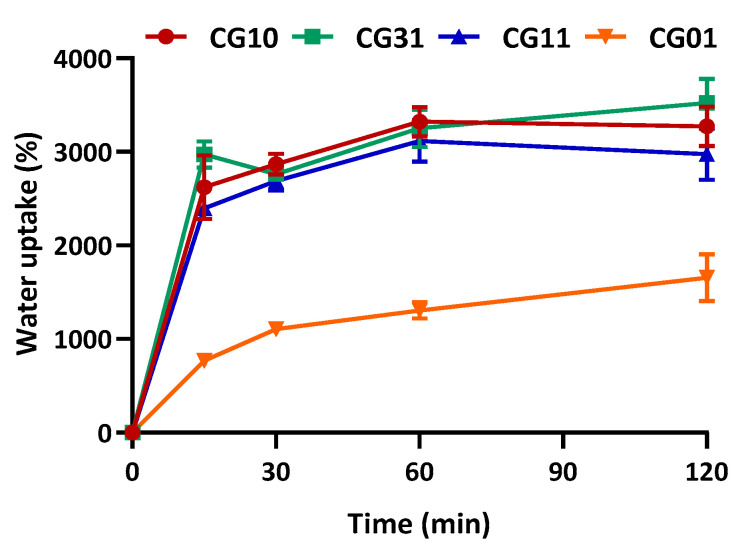
Water uptake of NGC tubular scaffolds with different chitosan/gelatin ratios (1:0, 3:1, 1:1, 0:1) as a function of time, performed in 10 mM PBS, pH 7.4, 25 °C. Data are reported as the mean values from *n* = 3 replicates, expressed as the mean value of the percentage of water uptake (± standard deviation).

The water uptake capability of natural polymer-based scaffolds is known to be strictly related to the inherent porosity and to the presence of a large number of functional groups capable of binding water. Therefore, the water uptake percentage of the NGCs, as assessed in 10 mM PBS (pH 7.4, 25 °C) and calculated according to Equation (1), is shown in Figure 6. The NGC scaffolds reached the maximum swelling capacity in 2 h of incubation in PBS solution, with a significant increase (*p* < 0.05) in the presence of chitosan.

**Figure 7:**
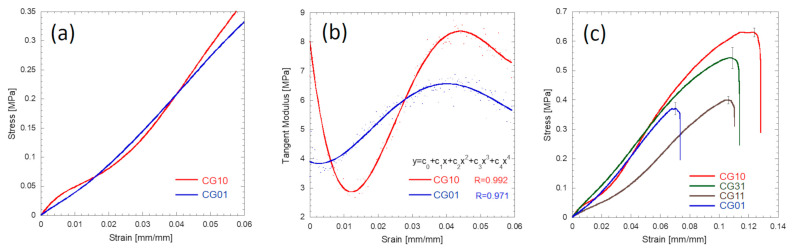
Mechanical behaviour of the CG10 and CG01 tubular specimens stretched at 1.0 mm/mm: typical stress vs. strain behaviour during the initial tensile stage (**a**); tangent modulus profiles during the initial tensile stage (**b**); typical stress vs. strain curves up to rupture for the investigated materials (**c**).

**Figure 8:**
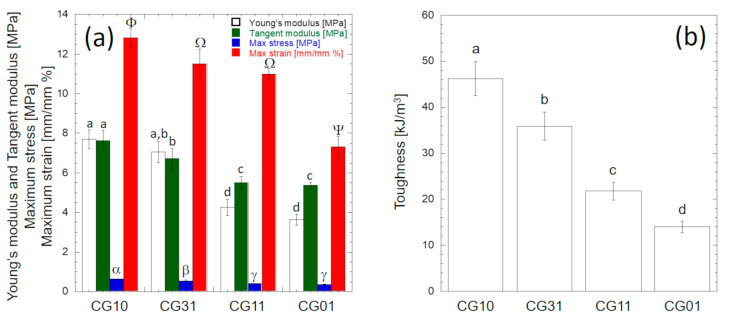
Mechanical properties of chitosan/gelatin tubular conduits: (**a**) bar plot of mechanical properties detected on stress vs. strain curves according to Figure 2g; (**b**) bar plot of toughness values computed by integrating the stress vs. strain curves (Figure 7c) up to the breaking point. The error bars represent the standard deviations. Different letters reported on each bar denote significant statistical difference according to Tukey’s statistical test (*p* < 0.05).

The mechanical behaviour of different 3D scaffolds was also carefully investigated and correlated with the morphological data (Figure 7 and Figure 8). The chitosan and gelatin porous scaffolds showed non-linear tensile behaviour. This behaviour is commonly observed for gelatin and chitosan porous structures undergoing tensile testing [54]. Figure 7a shows that at the beginning of the stretching, a steep region was found, and this behaviour was particularly evident for the chitosan sample. Figure 7b reports the profile of the tangent modulus of chitosan 3% and gelatin 3% computed by considering the derivative function of the stress vs. strain curves. The initial value of the tangent modulus profile corresponded to Young’s modulus, where the tangent modulus decreased down to a minimum and then increased up to a maximum value. A fourth-order polynomial function described the behaviour of the tangent modulus. Stress vs. strain diagrams for all the investigated materials are shown in Figure 7c.

Figure 8 shows the investigated mechanical properties determined according to Figure 2g. The overall results obtained from the analysis of variance for each mechanical property reported in Table 4 suggest that at the 0.05 level, the population mean of each mechanical property was significantly different. The stiffness (e.g., Young’s modulus or tangent modulus), the strength (i.e., maximum stress) and the compliance (i.e., maximum strain) values of CG10 were significantly higher (*p* < 0.05) than those of CG01 (Figure 8a).

In particular, according to Tukey’s post hoc test, the Young’s modulus and maximum tangent modulus values of CG10 (7.70 ± 0.49 MPa and 7.62 ± 0.50 MPa, respectively) were significantly higher (*p* < 10^−7^) than those of CG01 (3.63 ± 0.27 MPa and 5.37 ± 0.15 MPa, respectively). The Young’s modulus values computed for CG10 and CG01 were consistent with the values reported by Tseng et al. for similar structures in the dry state [55]. No statistically significant difference was found between the Young’s modulus and the maximum tangent modulus of CG10 (*p* = 0.21), while the maximum tangent modulus of CG01 was significantly higher (*p* < 10^−7^) than the Young’s modulus (Figure 8a). The strength of CG10 (0.634 ± 0.015 MPa) was significantly higher (*p* < 0.05) than the strengths of CG31 (0.543 ± 0.036 MPa), CG11 (0.396 ± 0.013 MPa) and CG01 (0.367 ± 0.021 MPa) (Figure 8a). However, no significant difference existed between the CG01 and CG11 strength values (*p* = 0.23). The strength value measured for CG10 was slightly higher than that reported by Ikeda et al. [56], while it was slightly lower than the strength reported for the chitosan scaffolds made by Kumar et al. [57]. The compliance value of CG01 (7.30 ± 0.24%) was significantly lower (*p* < 0.05) than the compliance values measured for CG10 (12.81 ± 0.21%), CG31 (11.59 ± 0.72%) and CG11 (10.19 ± 0.27%). Figure 8b reports the toughness values of the investigated porous scaffolds computed by integrating the stress vs. strain curves (Figure 7c) up to the breaking point. A remarkable significant increase (*p* < 0.05) was observed as the amount of chitosan increased. In particular, the toughness of the CG10 porous scaffold (46.2 kJ/m^3^) was 330% higher than the CG01 scaffold (14.0 kJ/m^3^).

Overall, these results clearly indicate that the addition of gelatin allowed for modulating the mechanical properties of the tubes, which gradually reduced the stiffness and scaffold toughness as the gelatin content increased, in agreement with other experimental studies on similar systems [58]. Moreover, these properties can be comparable with those of similar devices fabricated via other processing techniques (i.e., electrospinning, melt spinning), despite a significant increase in porosity (Table 5). These results can be relevant to overcoming typical suture problems that were recorded in the currently commercialized conduits made of collagen [59].

**Figure 9:**
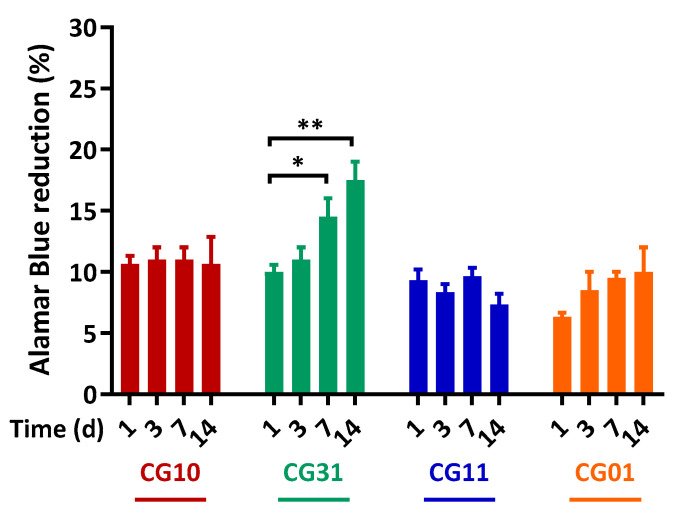
SH-SY5Y proliferation on the NGC tubular scaffolds. Time-dependent proliferation of SH-SY5Y after 1, 3, 7 and 14 days, as determined by an Alamar Blue^®^ assay. Data are the mean values from *n* = 3 biological replicates, expressed as a percentage of the Alamar Blue^®^ reduction (±standard deviation). Statistical analysis of the variance of the means was assessed by a two-way ANOVA and Bonferroni’s post hoc test [* *p* < 0.05, ** *p* < 0.01].

Finally, SH-SY5Y neuroblastoma cells were used as an in vitro model for axonal regeneration and seeded into NGCs scaffolds. The capability of NGC tubular scaffolds to support/promote cell proliferation was evaluated through a direct cytotoxicity test by using the Alamar Blue^®^ assay. Figure 9 shows SH-SY5Y cell proliferation up to 14 days of culture in NGC tubular scaffolds. The CG10, CG11 and CG01 scaffolds proved their capability to support SH-SY5Y survival over the culture time. Noteworthy, a significant influence on cell proliferation was noticed for CG31 (7 days—* *p* < 0.05, and 14 days—** *p* < 0.01) related to their peculiar morphological and mechanical features. In particular, gelatin embedded into the CG31 ensured the right balance between mechanical and chemical signals that was able to more efficiently promote cell proliferation on the porous scaffold.

In summary, overcoming the traditional idea of a nerve conduit working as a passive device—i.e., it is able to solely physically connect the injured stumps—the proposed systems may provide a controlled pattern of morphological, biochemical and biomechanical cues that are suitable to more actively support the biological processes involved during nerve regeneration. It is well known that the activity of newly formed neurites can be distinguished into two temporal phases: initial protrusion and axon cone formation triggered by intracellular calcium penetration [67] and late neurites that mainly grow regulated by the synergic effect of signalling proteins (i.e., including extracellular signal-related kinases [68]) and transcription factor signal transduction [69]. Accordingly, future advances in the research and innovation of chitosan-based nerve conduits should be addressed to optimize the process design and properties to more accurately match the spatiotemporal development of nerve tissue during in vivo regeneration. For this purpose, an emerging route is involving the implementation of additive manufacturing strategies to integrate micro- and nano-architectures with bioactive and electroactive cues that are able to selectively address—directly by chemo-physical guidance, or indirectly by molecular transport and signalling—the different phases of axon growth (e.g., sprouting, extension) up until the formation of new functional tissue with appropriate myelination and surrounding muscle reinnervation [70]. Recently, Takeya et al. proposed a technique to produce a double-layered nerve conduit composed of an outer layer of chitosan hydrogel and an inner layer of collagen hydrogel to better encapsulate cells to enhance peripheral nerve restoration [45].

**Figure 10:**
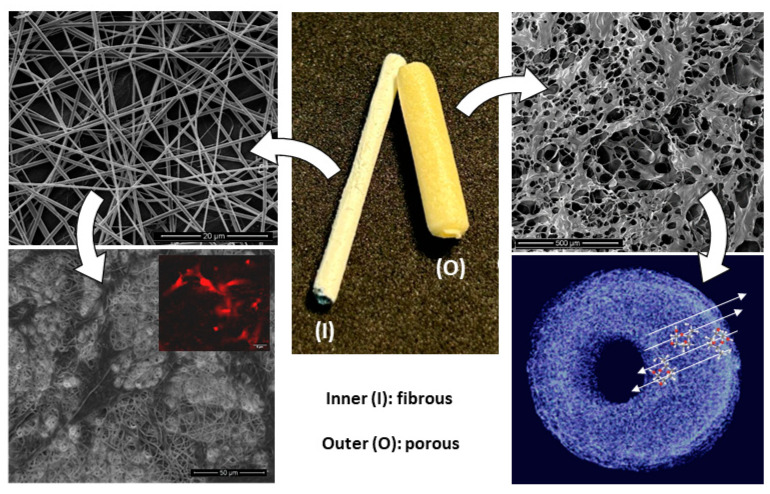
Design of bi-layered nerve conduits: (I) gelatin-based electrospun fibers to promote cell interaction and (O) chitosan-based porous shell to support molecular transport and signalling.

Inspired by this idea, a new strategy to fabricate nerve conduits could be quickly obtained by a combination with the electrospinning technique to design innovative bi-layered systems. The proposed idea—schematized in Figure 10—basically involved collecting gelatin-added fibers onto a metallic rod composing the mould before the chitosan solution casting. During the freeze-drying step, chitosan solution only slightly intruded through the fibrous mesh, thus forming a stable interface, and did not substantially alter the porosity.

This produced a bi-layer system with several improvements with respect to the standard porous tubes as follows: the inner layer composed of gelatin-based—randomly organized or preferentially aligned—fibers could guide neurons and non-neuronal cells just present in the area that comprised the two end parts of the peripheral nerves and supported the biological events connected to the nerve regeneration, as confirmed by different in vitro and in vivo studies in the literature on different nerve cells (i.e., Schwann cells [71], dorsal root ganglial [72], glial [73], astrocytes [74] or mesenchymal cells under neurogenic stimuli [75]). Indeed, the full interconnection of the porous structure formed by the electrospun fibers promoted and better guided nerve ingrowth—with respect to non-porous conduits—which led to more efficient oxygenation of the axons and nutrient exchange from the lumen to the outside in the absence of the distal nerve stump [11].

In this context, gelatin embedded into the fibrous network could more efficiently support cell adhesion and proliferation due to the retention of the triple helical structure and RGD-like sequences after the hydrolysis of the collagen [76,77,78]. Meanwhile the outer shell—composed of a porous chitosan/gelatin scaffold—could support the molecular transport by an efficient diffusion of nutrients and oxygen to the surrounding cells, and thus, facilitate cell differentiation and regeneration processes, as reported in previous works on similar scaffolds [13,79]. Moreover, recent experimental evidence also confirmed an active role of a porous chitosan network on supporting newly formed vascularization [80] and angiogenesis in the presence of gelatin [81] that is crucial to ensure an appropriate blood supply and oxygenation to the nerve cells.

## 4. Conclusions

In this work, a novel cost-effective method was proposed to engineer highly re-producible 3D porous scaffolds that consisted of a combination of chitosan and gelatin with different ratios to be used as nerve guide conduits. In particular, it was demonstrated that the presence of gelatin could affect the scaffold performance in terms of morphological (e.g., porosity, pore size) and mechanical properties. In detail, a slight increase in the porosity percentage was detected for CG01 NGCs, with a tendency to form 3D porous networks with more irregular and asymmetric structures and different pore aspect ratios. More interestingly, the addition of gelatin allowed for modulating the mechanical responses of the 3D scaffolds by gradually reducing the stiffness and scaffold toughness.

In this view, the proposed preparation method is highly versatile and offers different solutions to tailor the final properties of the device. First, it can be used to handle a polymer solution with different phase ratios to form 3D scaffolds with modular mechanical properties. This can allow for developing devices that are more affordable for clinical use due to their better capability to be sutured at the two end parts of the nerve lesion. Second, it can be widely customized for the fabrication of hollow porous tubes with different lengths, thus giving the opportunity to develop devices suitable for the repair of defects larger than 3 cm—this is still a relevant challenge in current nerve surgery. To date, experimental evidence on commercialized tubes with similar architecture have demonstrated to be unable to recover complete nerve function, showing a relevant performance decay, in comparison with autografts. This is generally due to the inability to form inner luminal structures coupled with blood clot breaking—mandatory to support the alignment of axons and Schwann cells during the regeneration process—so contributing to the implant failure, especially in the case of a long-gap treatment [82,83]. To some extent, the proposed technological solution can help to bridge this gap by providing more efficient customization of the process manufacturing by offering an easy-to-handle route to fabricate porous tubes endowed with multiple sets of biological, chemical and/or morphological signals suitable to reproduce the nerve microenvironment. In this view, the idea of combining it with other processing techniques (e.g., electrospinning, 3D printing) could suggest a valid strategy to design innovative bi-layered/multi-layered systems with an improved inner cell interface and functional features in terms of molecular transport, fluid permeability and electrical conductivity.

## Figures and Tables

**Table 1:** NGC tubular scaffolds: compositions and process conditions.

Type	CG Ratio	GP (% w_GP_/w_CG_)	T_Freezing_ (°C)	t_Drying_ (h)
CG10	1:0	0.5	−18	24
CG31	3:1	0.5	−18	24
CG11	1:1	0.5	−18	24
CG01	0:1	0.5	−18	24

**Table 2:** Summary of operative parameters used for microcomputed tomography analysis.

Field of View (FOV, mm^2^)	Acquisition Resolution (µm/pixel)	X-ray Detector Position (mm)	Exposure Time (s)	Range of Sample Rotation Angle (Degrees)	Number of Projections	Scan Time (min)
2.662 × 2.662	1.3	2	5	180	600	70
10.65 × 10.65	5.2	2	5	180	600	70

**Table 3:** Summary of porosity data.

Sample	Porosity (%) *	Porosity (%) ^§^	Pore Size (μm) ^§^		Aspect Ratio ^§^ α = a/b
			a	b	
CG10	94.07 ± 1.04	94.95 ± 0.56	103.47 ± 23.17	134.16 ± 26.10	0.79 ± 0.17
CG31	94.03 ± 1.02	95.35 ± 1.14	96.74 ± 27.07	121.35 ± 30.33	0.80 ± 0.15
CG11	95.01 ± 1.21	95.70 ± 0.49	67.86 ± 18.89	90.62 ± 23.99	0.76 ± 0.14
CG01	97.23 ± 1.15	96.86 ± 0.31	186.62 ± 58.96	276.65 ± 90.07	0.70 ± 0.19

* Gravimetric method; ^§^ micro-CT analysis.

**Table 4:** Analysis of the variance results for each determined mechanical property. At the 0.05 level, the population means of each mechanical property were significantly different.

		DF	Sum of Squares	Mean Square	F Value	Prob > F
Young’s modulus	Model	3	61.22204	20.40735	90.16932	3.07862 × 10^−10^
	Error	16	3.62116	0.22632		
	Total	19	64.8432			
Tangent modulus	Model	3	16.1404	5.38013	33.06527	4.32898 × 10^−7^
	Error	16	2.6034	0.16271		
	Total	19	18.7438			
Maximum stress	Model	3	0.2367	0.0789	144.38634	8.51763 × 10^−12^
	Error	16	0.00874	5.4645 × 10^−4^		
	Total	19	0.24544			
Maximum strain	Model	3	84.255	28.085	100.81163	1.32562 × 10^−10^
	Error	16	4.45742	0.27859		
	Total	19	88.71242			

**Table 5:** Comparison between different features of NGC analogues prepared via different approaches (i.e., freeze drying, electrospinning, thermally induced phase separation) compared with native sciatic nerves and decellularized nerves.

NGCs Fabrication Technique	Material	Porosity (%)	Mechanical Data	Ref.
Native sciatic nerve	Rat source	N/A	Tensile strength = 6.5–11.7 MPa Elastic modulus = 0.58 MPa	[60]
Acellularization	F344 rat sciatic nerves	N/A	Elastic modulus = 0.58 ± 0.16 MPa Tensile stress = 0.78 ± 0.28 MPa	[60]
Freeze drying	Chitosan	-	Tensile stress = 0.75–0.95 MPa at 5.8% elongation	[61]
Freeze drying	Chitosan/collagen	-	Tensile modulus = 0.087 ± 0.007 MPa	[62]
Molding/freeze drying	Genipin-crosslinked chitosan/gelatin	94.0–97.0	Elastic modulus = 3.63–7.70 MPa Tensile strength = 0.367–0.634 MPa Maximum strain = 7.30–12.81% Toughness = 14.0–46.2 kJ/m^3^	Our data
Molding/freeze drying	Chitosan/chitosan yarns	-	Tensile strength = 3.69 ± 0.64 MPa	[63]
Molding/freeze drying	Genipin-crosslinked gelatin	90.8 ± 0.9%	Maximum tensile force = 0.03 ± 0.01 kN	[64]
Electrospinning	Chitosan/PEO	0.180 ± 0.02 g/cm^3^ (density)	Elastic modulus = 0.589 MPa	[65]
Melt spinning	Chitosan/crosslinked carboxymethyl chitosan	69.8%	Elastic modulus = 3.59 MPa Max load = 10.83 N	[66]

## Data Availability

The data used in this study are available in this article.

## Supplementary Materials

The following supporting information can be downloaded from https://www.mdpi.com/article/10.3390/polym16202893/s1: Figure S1. Ninhydrin assay. Ninhydrin absorbance recorded in the supernatant after 20 min of incubation with scaffolds with different chitosan/gelatin weight ratios and corresponding representative pictures of scaffolds incubated with the ninhydrin solution showing the variation in the colour due to the released gelatin.

## References

[1] Lutzweiler G., Ndreu Halili A., Engin Vrana N. (2020). The Overview of Porous, Bioactive Scaffolds as Instructive Biomaterials for Tissue Regeneration and Their Clinical Translation. Pharmaceutics.

[2] Poh P.S.P., Valainis D., Bhattacharya K., Van Griensven M., Dondl P. (2019). Optimization of Bone Scaffold Porosity Distributions. Sci. Rep..

[3] Guarino V., Guaccio A., Netti P.A., Ambrosio L. (2010). Image Processing and Fractal Box Counting: User-Assisted Method for Multi-Scale Porous Scaffold Characterization. J. Mater. Sci. Mater. Med..

[4] Madaghiele M., Sannino A., Yannas I.V., Spector M. (2008). Collagen-Based Matrices with Axially Oriented Pores. J. Biomed. Mater. Res. Part A Off. J. Soc. Biomater. Jpn. Soc. Biomater. Aust. Soc. Biomater. Korean Soc. Biomater..

[5] Wang Y.-F., Barrera C.M., Dauer E.A., Gu W., Andreopoulos F., Huang C.-Y.C. (2017). Systematic Characterization of Porosity and Mass Transport and Mechanical Properties of Porous Polyurethane Scaffolds. J. Mech. Behav. Biomed. Mater..

[6] Guarino V., Causa F., Ambrosio L. (2007). Porosity and Mechanical Properties Relationship in PCL Porous Scaffolds. J. Appl. Biomater. Biomech..

[7] Offeddu G.S., Mohee L., Cameron R.E. (2020). Scale and Structure Dependent Solute Diffusivity within Microporous Tissue Engineering Scaffolds. J. Mater. Sci. Mater. Med..

[8] Ambekar R.S., Kandasubramanian B. (2019). Progress in the Advancement of Porous Biopolymer Scaffold: Tissue Engineering Application. Ind. Eng. Chem. Res..

[9] Flores-Jiménez M.S., Garcia-Gonzalez A., Fuentes-Aguilar R.Q. (2023). Review on Porous Scaffolds Generation Process: A Tissue Engineering Approach. ACS Appl. Bio Mater..

[10] Subramanian A., Krishnan U.M., Sethuraman S. (2009). Development of Biomaterial Scaffold for Nerve Tissue Engineering: Biomaterial Mediated Neural Regeneration. J. Biomed. Sci..

[11] Guarino V., Cirillo V., Ambrosio L. (2016). Bicomponent Electrospun Scaffolds to Design Extracellular Matrix Tissue Analogs. Expert Rev. Med. Devices.

[12] Gao S., Chen X., Lu B., Meng K., Zhang K.-Q., Zhao H. (2023). Recent Advances on Nerve Guide Conduits Based on Textile Methods. Smart Mater. Med..

[13] Farokhi M., Mottaghitalab F., Shokrgozar M.A., Kaplan D.L., Kim H.-W., Kundu S.C. (2017). Prospects of Peripheral Nerve Tissue Engineering Using Nerve Guide Conduits Based on Silk Fibroin Protein and Other Biopolymers. Int. Mater. Rev..

[14] Sarker M.D., Naghieh S., McInnes A.D., Schreyer D.J., Chen X. (2018). Regeneration of Peripheral Nerves by Nerve Guidance Conduits: Influence of Design, Biopolymers, Cells, Growth Factors, and Physical Stimuli. Prog. Neurobiol..

[15] Harley B.A., Hastings A.Z., Yannas I.V., Sannino A. (2006). Fabricating Tubular Scaffolds with a Radial Pore Size Gradient by a Spinning Technique. Biomaterials.

[16] Chang A., Yannas I.V., Perutz S., Loree H., Sethi R.R., Krarup C., Norregaard T.V., Zervas N.T., Silver J. (1990). Electrophysiological Study of Recovery of Peripheral Nerves Regenerated by a Collagen-Glycosaminoglycan Copolymer Matrix. Progress in Biomedical Polymers.

[17] Salvatore L., Madaghiele M., Parisi C., Gatti F., Sannino A. (2014). Crosslinking of Micropatterned Collagen-Based Nerve Guides to Modulate the Expected Half-Life. J. Biomed. Mater. Res. Part A.

[18] Cerri F., Salvatore L., Memon D., Boneschi F.M., Madaghiele M., Brambilla P., Del Carro U., Taveggia C., Riva N., Trimarco A. (2014). Peripheral Nerve Morphogenesis Induced by Scaffold Micropatterning. Biomaterials.

[19] Mligiliche N.L., Tabata Y., Ide C. (1999). Nerve Regeneration through Biodegradable Gelatin Conduits in Mice. East Afr. Med. J..

[20] Tian L., Prabhakaran M.P., Ramakrishna S. (2015). Strategies for Regeneration of Components of Nervous System: Scaffolds, Cells and Biomolecules. Regen. Biomater..

[21] Boecker A., Däschler S.C., Kneser U., Harhaus L. (2019). Relevance and Recent Developments of Chitosan in Peripheral Nerve Surgery. Front. Cell. Neurosci..

[22] Liu H., Wen W., Hu M., Bi W., Chen L., Liu S., Chen P., Tan X. (2013). Chitosan Conduits Combined with Nerve Growth Factor Microspheres Repair Facial Nerve Defects. Neural Regen. Res..

[23] Zhang M., An H., Zhang F., Jiang H., Wan T., Wen Y., Han N., Zhang P. (2023). Prospects of Using Chitosan-Based Biopolymers in the Treatment of Peripheral Nerve Injuries. Int. J. Mol. Sci..

[24] Böcker A., Aman M., Kneser U., Harhaus L., Siemers F., Stang F. (2022). Closing the Gap: Bridging Peripheral Sensory Nerve Defects with a Chitosan-Based Conduit a Randomized Prospective Clinical Trial. J. Pers. Med..

[25] Itai S., Suzuki K., Kurashina Y., Kimura H., Amemiya T., Sato K., Nakamura M., Onoe H. (2020). Cell-Encapsulated Chitosan-Collagen Hydrogel Hybrid Nerve Guidance Conduit for Peripheral Nerve Regeneration. Biomed. Microdevices.

[26] Li R., Liu H., Huang H., Bi W., Yan R., Tan X., Wen W., Wang C., Song W., Zhang Y. (2018). Chitosan Conduit Combined with Hyaluronic Acid Prevent Sciatic Nerve Scar in a Rat Model of Peripheral Nerve Crush Injury. Mol. Med. Rep..

[27] Chang J.-Y., Lin J.-H., Yao C.-H., Chen J.-H., Lai T.-Y., Chen Y.-S. (2007). In Vivo Evaluation of a Biodegradable EDC/NHS-Cross-Linked Gelatin Peripheral Nerve Guide Conduit Material. Macromol. Biosci..

[28] Chiono V., Pulieri E., Vozzi G., Ciardelli G., Ahluwalia A., Giusti P. (2008). Genipin-Crosslinked Chitosan/Gelatin Blends for Biomedical Applications. J. Mater. Sci. Mater. Med..

[29] Guarino V., Galizia M., Alvarez-Perez M., Mensitieri G., Ambrosio L. (2015). Improving Surface and Transport Properties of Macroporous Hydrogels for Bone Regeneration. J. Biomed. Mater. Res. Part A.

[30] Guarino V., Lewandowska M., Bil M., Polak B., Ambrosio L. (2010). Morphology and Degradation Properties of PCL/HYAFF11®composite Scaffolds with Multi-Scale Degradation Rate. Compos. Sci. Technol..

[31] De Santis R., D’Amora U., Russo T., Ronca A., Gloria A., Ambrosio L. (2015). 3D Fibre Deposition and Stereolithography Techniques for the Design of Multifunctional Nanocomposite Magnetic Scaffolds. J. Mater. Sci. Mater. Med..

[32] Russo T., Gloria A., D’Antò V., D’Amora U., Ametrano G., Bollino F., De Santis R., Ausanio G., Catauro M., Rengo S. (2010). Poly(∊-Caprolactone) Reinforced with Sol-Gel Synthesized Organic-Inorganic Hybrid Fillers as Composite Substrates for Tissue Engineering. J. Appl. Biomater. Biomech..

[33] (2021). Biological Evaluation of Medical Devices Part 12: Sample Preparation and Reference Materials.

[34] Hosseinkhani H., Hiraoka Y., Li C.-H., Chen Y.-R., Yu D.-S., Hong P.-D., Ou K.-L. (2013). Engineering Three-Dimensional Collagen-IKVAV Matrix to Mimic Neural Microenvironment. ACS Chem. Neurosci..

[35] Chen P., Cescon M., Megighian A., Ronaldo P. (2014). Collagen VI Regulates Peripheral Nerve Myelination and Function. FASEB J..

[36] Boni R., Ali A., Shavandi A., Clarkson A.N. (2018). Current and Novel Polymeric Biomaterials for Neural Tissue Engineering. J. Biomed. Sci..

[37] Li X., Zhang X., Hao M., Wang D., Jiang Z., Sun L., Gao Y., Jin Y., Lei P., Zhuo Y. (2022). The Application of Collagen in the Repair of Peripheral Nerve Defect. Front. Bioeng. Biotechnol..

[38] Aranaz I., Alcántara A.R., Civera M.C., Arias C., Elorza B., Heras Caballero A., Acosta N. (2021). Chitosan: An Overview of Its Properties and Applications. Polymers.

[39] Yin Y., Li Z., Sun Y., Yao K. (2005). A Preliminary Study on Chitosan/Gelatin Polyelectrolyte Complex Formation. J. Mater. Sci..

[40] Manzari-Tavakoli A., Tarasi R., Sedghi R., Moghimi A., Niknejad H. (2020). Fabrication of Nanochitosan Incorporated Polypyrrole/Alginate Conducting Scaffold for Neural Tissue Engineering. Sci. Rep..

[41] Meek M.F., Coert J.H. (2008). US Food and Drug Administration/Conformit Europe-Approved Absorbable Nerve Conduits for Clinical Repair of Peripheral and Cranial Nerves. Ann. Plast. Surg..

[42] Zhao Y., Wang Y., Gong J., Yang L., Niu C., Ni X., Wang Y., Peng S., Gu X., Sun C. (2017). Chitosan Degradation Products Facilitate Peripheral Nerve Regeneration by Improving Macrophage-Constructed Microenvironments. Biomaterials.

[43] He B., Tao H.-Y., Liu S.-Q. (2014). Neuroprotective Effects of Carboxymethylated Chitosan on Hydrogen Peroxide Induced Apoptosis in Schwann Cells. Eur. J. Pharmacol..

[44] Marcol W., Larysz-Brysz M., Kucharska M., Niekraszewicz A., Slusarczyk W., Kotulska K., Wlaszczuk P., Wlaszczuk A., Jedrzejowska-Szypulka H., Lewin-Kowalik J. (2011). Reduction of Post-Traumatic Neuroma and Epineural Scar Formation in Rat Sciatic Nerve by Application of Microcrystallic Chitosan. Microsurgery.

[45] Takeya H., Itai S., Kimura H., Kurashina Y., Amemiya T., Nagoshi N., Iwamoto T., Sato K., Shibata S., Matsumoto M. (2023). Schwann Cell-Encapsulated Chitosan-Collagen Hydrogel Nerve Conduit Promotes Peripheral Nerve Regeneration in Rodent Sciatic Nerve Defect Models. Sci. Rep..

[46] Sogias I.A., Khutoryanskiy V.V., Williams A.C. (2010). Exploring the Factors Affecting the Solubility of Chitosan in Water. Macromol. Chem. Phys..

[47] Vinsova J., Vavrikova E. (2011). Chitosan Derivatives with Antimicrobial, Antitumour and Antioxidant Activities-a Review. Curr. Pharm. Des..

[48] Scialla S., Gullotta F., Izzo D., Palazzo B., Scalera F., Martin I., Sannino A., Gervaso F. (2022). Genipin-Crosslinked Collagen Scaffolds Inducing Chondrogenesis: A Mechanical and Biological Characterization. J. Biomed. Mater. Res. Part A.

[49] Roy S., Rhim J.-W. (2022). Genipin-Crosslinked Gelatin/Chitosan-Based Functional Films Incorporated with Rosemary Essential Oil and Quercetin. Materials.

[50] Queiroz M.F., Teodosio Melo K.R., Sabry D.A., Sassaki G.L., Rocha H.A.O. (2014). Does the Use of Chitosan Contribute to Oxalate Kidney Stone Formation?. Mar. Drugs.

[51] Zhang Y., Wang Q.-S., Yan K., Qi Y., Wang G.-F., Cui Y.-L. (2016). Preparation, Characterization, and Evaluation of Genipin Crosslinked Chitosan/Gelatin Three-Dimensional Scaffolds for Liver Tissue Engineering Applications. J. Biomed. Mater. Res. Part A.

[52] Das M.P., Suguna P.R., Prasad K., Vijaylakshmi J.V., Renuka M. (2017). Extraction and Characterization of Gelatin: A Functional Biopolymer. Int. J. Pharm. Pharm. Sci..

[53] Grenier J., Duval H., Barou F., Lv P., David B., Letourneur D. (2019). Mechanisms of Pore Formation in Hydrogel Scaffolds Textured by Freeze-Drying. Acta Biomater..

[54] Suo H., Zhang D., Yin J., Qian J., Wu Z.L., Fu J. (2018). Interpenetrating Polymer Network Hydrogels Composed of Chitosan and Photocrosslinkable Gelatin with Enhanced Mechanical Properties for Tissue Engineering. Mater. Sci. Eng. C.

[55] Tseng H.-J., Tsou T.-L., Wang H.-J., Hsu S. (2013). Characterization of Chitosan--Gelatin Scaffolds for Dermal Tissue Engineering. J. Tissue Eng. Regen. Med..

[56] Ikeda T., Ikeda K., Yamamoto K., Ishizaki H., Yoshizawa Y., Yanagiguchi K., Yamada S., Hayashi Y. (2014). Fabrication and Characteristics of Chitosan Sponge as a Tissue Engineering Scaffold. BioMed Res. Int..

[57] Kumar P., Dehiya B.S., Sindhu A. (2017). Comparative Study of Chitosan and Chitosan--Gelatin Scaffold for Tissue Engineering. Int. nano Lett..

[58] Pezeshki-Modaress M., Rajabi-Zeleti S., Zandi M., Mirzadeh H., Sodeifi N., Nekookar A., Aghdami N. (2014). Cell-Loaded Gelatin/Chitosan Scaffolds Fabricated by Salt-Leaching/Lyophilization for Skin Tissue Engineering: In Vitro and In Vivo Study. J. Biomed. Mater. Res. Part A.

[59] Clifford A.L., Klifto C.S., Li N.Y. (2024). Nerve Coaptation in 2023: Adjuncts to Nerve Repair Beyond Suture. J. Hand Surg. Glob. Online.

[60] Borschel G.H., Kia K.F., Kuzon W.M., Dennis R.G. (2003). Mechanical Properties of Acellular Peripheral Nerve. J. Surg. Res..

[61] Yang Y., Gu X., Tan R., Hu W., Wang X., Zhang P., Zhang T. (2004). Fabrication and Properties of a Porous Chitin/Chitosan Conduit for Nerve Regeneration. Biotechnol. Lett..

[62] Hu X., Huang J., Ye Z., Xia L., Li M., Lv B., Shen X., Luo Z. (2009). A Novel Scaffold with Longitudinally Oriented Microchannels Promotes Peripheral Nerve Regeneration. Tissue Eng. Part A.

[63] Wang A., Ao Q., Wei Y., Gong K., Liu X., Zhao N., Gong Y., Zhang X. (2007). Physical Properties and Biocompatibility of a Porous Chitosan-Based Fiber-Reinforced Conduit for Nerve Regeneration. Biotechnol. Lett..

[64] Chang J.-Y., Ho T.-Y., Lee H.-C., Lai Y.-L., Lu M.-C., Yao C.-H., Chen Y.-S. (2009). Highly Permeable Genipin-Cross-Linked Gelatin Conduits Enhance Peripheral Nerve Regeneration. Artif. Organs.

[65] Garcia Garcia C.E., Bossard F., Rinaudo M. (2021). Electrospun Biomaterials from Chitosan Blends Applied as Scaffold for Tissue Regeneration. Polymers.

[66] Zhang Y., Jiang Z., Wang Y., Xia L., Yu S., Li H., Zhang W., Liu W., Shao K., Han B. (2022). Nerve Regeneration Effect of a Composite Bioactive Carboxymethyl Chitosan-Based Nerve Conduit with a Radial Texture. Molecules.

[67] Bradke F., Fawcett J.W., Spira M.E. (2012). Assembly of a New Growth Cone after Axotomy: The Precursor to Axon Regeneration. Nat. Rev. Neurosci..

[68] Ben-Yaakov K., Dagan S.Y., Segal-Ruder Y., Shalem O., Vuppalanchi D., Willis D.E., Yudin D., Rishal I., Rother F., Bader M. (2012). Axonal Transcription Factors Signal Retrogradely in Lesioned Peripheral Nerve. EMBO J..

[69] Martins R.S., Bastos D., Siqueira M.G., Heise C.O., Teixeira M.J. (2013). Traumatic Injuries of Peripheral Nerves: A Review with Emphasis on Surgical Indication. Arq. Neuropsiquiatr..

[70] Yan Y., Yao R., Zhao J., Chen K., Duan L., Wang T., Zhang S., Guan J., Zheng Z., Wang X. (2022). Implantable Nerve Guidance Conduits: Material Combinations, Multi-Functional Strategies and Advanced Engineering Innovations. Bioact. Mater..

[71] Lezcano M.F., Martínez-Rodríguez P., Godoy K., Hermosilla J., Acevedo F., Gareis I.E., Dias F.J. (2023). Exploring Schwann Cell Behavior on Electrospun Polyhydroxybutyrate Scaffolds with Varied Pore Sizes and Fiber Thicknesses: Implications for Neural Tissue Engineering. Polymers.

[72] Cirillo V., Clements B.A., Guarino V., Bushman J., Kohn J., Ambrosio L. (2014). A Comparison of the Performance of Mono-and Bi-Component Electrospun Conduits in a Rat Sciatic Model. Biomaterials.

[73] Puhl D.L., Funnell J.L., Nelson D.W., Gottipati M.K., Gilbert R.J. (2020). Electrospun Fiber Scaffolds for Engineering Glial Cell Behavior to Promote Neural Regeneration. Bioengineering.

[74] Saracino E., Cirillo V., Marrese M., Guarino V., Benfenati V., Zamboni R., Ambrosio L. (2021). Structural and Functional Properties of Astrocytes on PCL Based Electrospun Fibres. Mater. Sci. Eng. C.

[75] Cirillo V., Guarino V., Alvarez-Perez M.A., Marrese M., Ambrosio L. (2014). Optimization of Fully Aligned Bioactive Electrospun Fibers for “in Vitro” Nerve Guidance. J. Mater. Sci. Mater. Med..

[76] Alipour H., Alizadeh A., Azarpira N., Saudi A., Alavi O., Tanideh N., Dehghani F. (2022). Incorporating Fingolimod through Poly(Lactic-Co-Glycolic Acid) Nanoparticles in Electrospun Polyurethane/Polycaprolactone/Gelatin Scaffold: An in Vitro Study for Nerve Tissue Engineering. Polym. Adv. Technol..

[77] Pozzobon L.G., Sperling L.E., Teixeira C.E., Malysz T., Pranke P. (2021). Development of a Conduit of PLGA-Gelatin Aligned Nanofibers Produced by Electrospinning for Peripheral Nerve Regeneration. Chem. Biol. Interact..

[78] Liu S., Sun X., Wang T., Chen S., Zeng C., Xie G., Zhu Q., Liu X., Quan D. (2018). Nano-Fibrous and Ladder-like Multi-Channel Nerve Conduits: Degradation and Modification by Gelatin. Mater. Sci. Eng. C.

[79] Deng P., Chen F., Zhang H., Chen Y., Zhou J. (2022). Multifunctional Double-Layer Composite Hydrogel Conduit Based on Chitosan for Peripheral Nerve Repairing. Adv. Healthc. Mater..

[80] Wlaszczuk A., Marcol W., Kucharska M., Wawro D., Palen P., Lewin-Kowalik J. (2016). Poly(D,L-Lactide-Co-Glycolide) Tubes with Multifilament Chitosan Yarn or Chitosan Sponge Core in Nerve Regeneration. J. Oral Maxillofac. Surg..

[81] Wang G., Lu P., Qiao P., Zhang P., Cai X., Tang L., Qian T., Wang H. (2022). Blood Vessel Remodeling in Late Stage of Vascular Network Reconstruction Is Essential for Peripheral Nerve Regeneration. Bioeng. Transl. Med..

[82] Kasper M., Deister C., Beck F., Schmidt C.E. (2020). Bench-to-Bedside Lessons Learned: Commercialization of an Acellular Nerve Graft. Adv. Healthc. Mater..

[83] Gonzalez-Perez F., Cobianchi S., Geuna S., Barwig C., Freier T., Udina E., Navarro X. (2015). Tubulization with Chitosan Guides for the Repair of Long Gap Peripheral Nerve Injury in the Rat. Microsurgery.

