# Supplementary material for: An Easy-to-Handle Route for Bicomponent Porous Tubes Fabrication as Nerve Guide Conduits
Source: Polymers (Basel). 2024 Oct 14;16(20):2893. doi: 10.3390/polym16202893 (PMC11511187; doi:10.3390/polym16202893)
Supplement: Supplementary file 1 [file polymers-16-02893-s001.zip › polymers-3250227-supplementary.pdf]

polymers

MDPI

Supporting Information

An Easy-To-Handle Route for Bicomponent Porous Tubes Fabrication as Nerve Guide Conduits

Teresa Russo †, Stefania Scialla †, Marietta D'Albore, Iriczalli Cruz-Maya, Roberto De Santis * and Vincenzo Guarino *

Institute for Polymers, Composites and Biomaterials (IPCB), National Research Council of Italy (CNR), Mostra d'Oltremare, Pad. 20, V. le J.F. Kennedy 54, 80125 Napoli, Italy; teresa.russo@cnr.it (T.R.); stefania.scialla@cnr.it (S.S.); mariettadalbore@hotmail.it (M.D.); iriczalli.cruzmaya@ipcb.cnr.it (I.C.-M.)

* Correspondence: roberto.desantis@cnr.it (R.D.S.); vincenzo.guarino@cnr.it (V.G.)

† These authors contributed equally to this work.

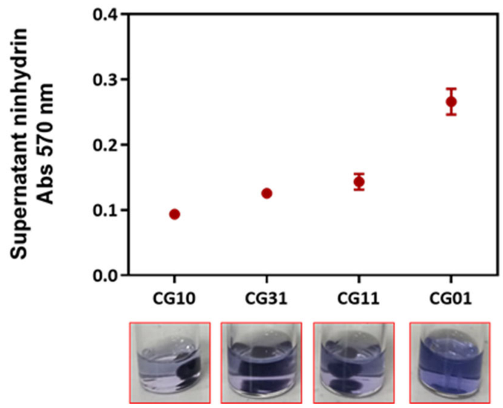

Samples	Abs $_{570nm}$	[Gelatin] $_{supernatant}$ (mg/mL)
**CG10**	0,093 ± 0,006	3.60 ± 0.39
**CG31**	0,125 ± 0,003	5.57 ± 0.21
**CG11**	0,143 ± 0,012	6.69 ± 0.75
**CG01**	0,266 ± 0,020	14.27 ± 1.24

Figure S1 Ninhydrin assay. Ninhydrin absorbance recorded in the supernatant after 20 min of incubation with scaffolds with different chitosan/gelatin weight ratios and corresponding representative pictures of scaffolds incubated with ninhydrin solution showing the variation of the colour due to released gelatin.
